# An Unusual Pattern of Burns Space Metastasis From Oral Squamous Cell Carcinoma Treated by Surgical Resection: A Case Report

**DOI:** 10.1155/crot/1895808

**Published:** 2026-07-15

**Authors:** Taisei Yasuda, Yasuhiro Ebihara, Yuichiro Enoki, Hirohisa Iwaki, Satoko Matsumura, Tomoko Yamazaki, Mitsuhiko Nakahira

**Affiliations:** ^1^ Department of Head and Neck Surgery, International Medical Center, Saitama Medical University, 1397-1 Yamane, Hidaka, Saitama, 350-1298, Japan, saitama-med.ac.jp

**Keywords:** altered lymphatic drainage pattern, bone invasion, Burns space, lingual cancer, suprasternal lymph node metastasis

## Abstract

**Introduction:**

The suprasternal space, termed Burns space, lies between the sternocleidomastoid and sternohyoid muscles. Although metastasis in this area frequently involves papillary thyroid carcinoma, it is rarely reported in head and neck squamous cell carcinoma.

**Case Presentation:**

We report a case of a 79‐year‐old man who underwent a partial glossectomy for tongue cancer, followed by neck dissection and radiotherapy. One year posttreatment, the patient developed suprasternal (Burns space) lymph node metastasis accompanied by bone invasion of the sternum and clavicle. An en bloc resection, which included the sternoclavicular joint, was performed, followed by reconstruction with a pectoralis major flap.

**Conclusion:**

To our knowledge, this is the first documented case of suprasternal lymph node metastasis with osseous extension from lingual cancer. A careful preoperative assessment of the suprasternal region is recommended, particularly in patients with a history of neck dissection (post‐ND).

## 1. Introduction

The suprasternal region (Burns space) is an anatomical area located between the sternocleidomastoid and sternohyoid muscles [1]. Although not typically included in standard neck dissection procedures, this region has recently gained attention as a potential site of lymph node metastasis, particularly in papillary thyroid carcinoma (PTC). Several studies have reported suprasternal lymph node involvement in PTC [2, 3]. However, lymphatic metastasis to this area from squamous cell carcinoma (SCC) of the oral cavity, particularly tongue SCC, is extremely rare. To our knowledge, no prior cases with concomitant osseous invasion have been reported.

Here, we describe a rare case of suprasternal lymph node metastasis with direct sternal and clavicular invasion occurring 1 year after neck dissection and radiotherapy for tongue cancer. This case highlights the potential for atypical lymphatic spread following surgical and radiotherapeutic intervention and underscores the need for preoperative assessment of nonconventional lymphatic drainage pathways.

## 2. Case Presentation

**FIGURE 1:**
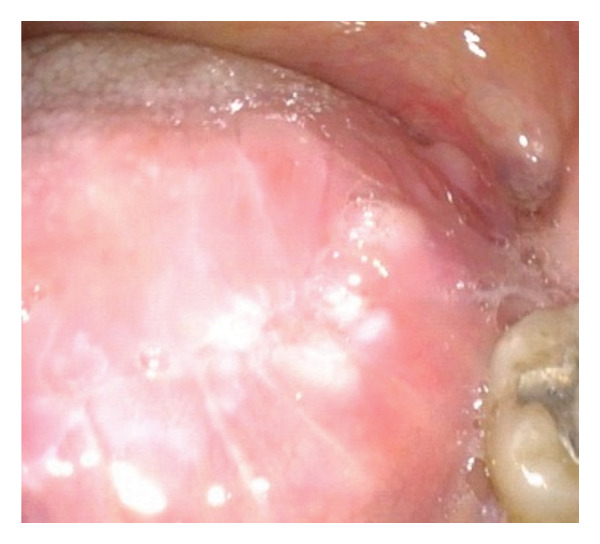
Left‐sided tongue squamous cell carcinoma, cT1N0M0. A whitish ulcerative lesion is observed on the left dorsal surface of the tongue.

A 79‐year‐old man presented with a left dorsal lingual mass in June 2022. His medical history included hypertension and a heavy smoking history (Brinkman index: 880). A biopsy confirmed SCC, and he was diagnosed with cT1N0M0 lingual cancer (Figure 1).

**FIGURE 2:**
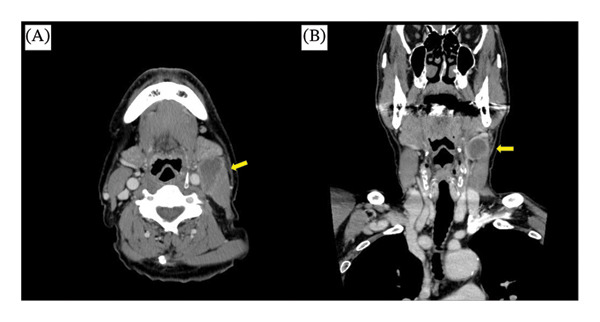
Delayed lymph node metastasis 6 months after partial glossectomy. (A) Axial and (B) coronal views. The arrows indicate metastasis.

In July 2022, the patient underwent a partial glossectomy with negative surgical margins. Six months postoperatively, contrast‐enhanced computed tomography revealed ipsilateral cervical lymph node metastasis (Figure 2).

**FIGURE 3:**
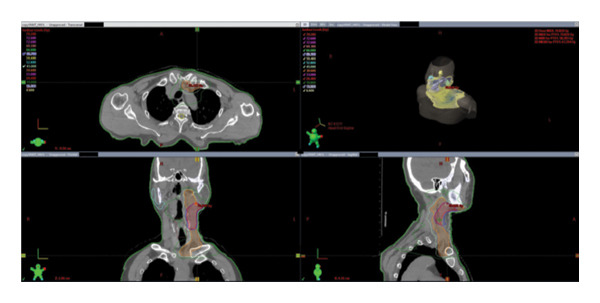
Radiation therapy dose distribution map. A total dose of 66 Gy was delivered using volumetric modulated arc therapy, primarily targeting Levels Ib and II.

A salvage‐modified radical neck dissection, including the internal jugular vein, was performed. Histopathological analysis identified a total of 25 removed lymph nodes. Among them, 12 lymph nodes (Levels IB and II) were positive for metastasis, whereas no metastasis was found in the other levels. Extranodal extension was observed in three metastatic lymph nodes at Levels IB and II. Due to the high risk of recurrence, postoperative radiotherapy was administered (66 Gy in 33 fractions) (Figure 3).

**FIGURE 4:**
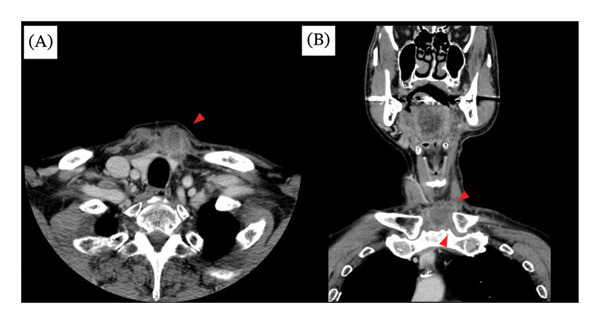
Suprasternal (Burns space) lymph nodes metastasis with bone invasion. (A) Axial and (B) coronal views. The arrowheads indicate metastasis.

In January 2024, approximately one year postradiotherapy, the patient developed suprasternal lymph node metastasis with sternal and clavicular invasion (Figure 4).

**FIGURE 5:**
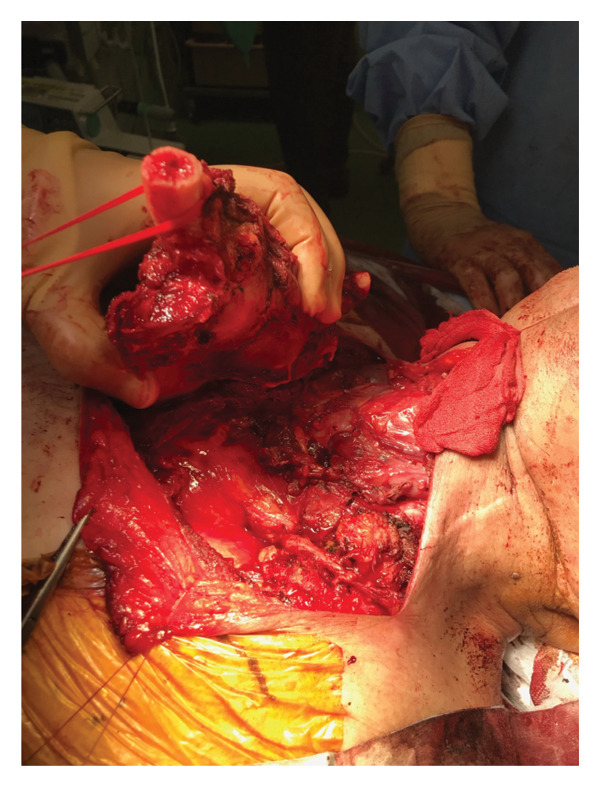
Intraoperative photograph showing en bloc resection of the lymph node metastasis invading the sternum together with the sternoclavicular joint. The left clavicle and the ribs were transected.

**FIGURE 6:**
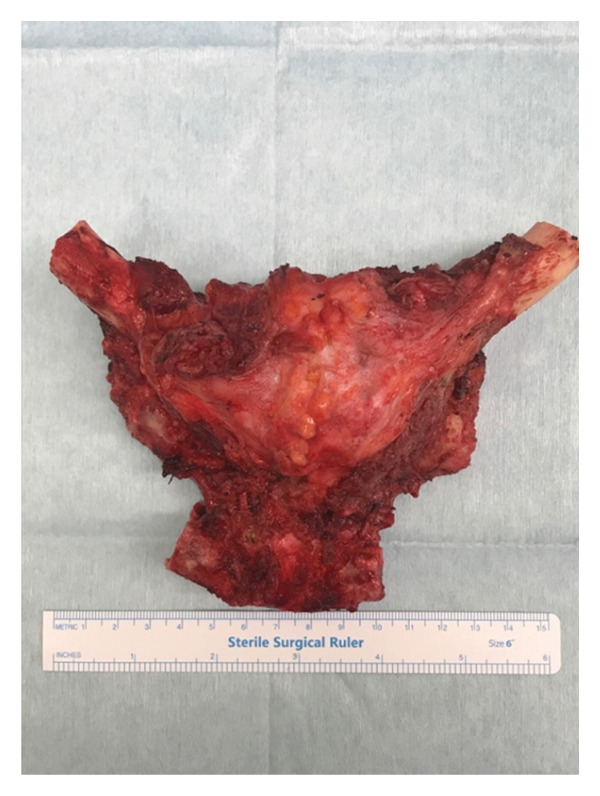
Resected tumor with the left clavicle and the ribs.

^18^F‐fluorodeoxyglucose positron emission tomography showed no mediastinal or hilar lymphadenopathy or distant metastases. Fine‐needle aspiration biopsy confirmed SCC. The patient underwent a combined surgical resection of the suprasternal lymph node metastasis invading the sternum and clavicle (Figure 5, 6).

Intraoperatively, the bilateral infrahyoid and sternocleidomastoid muscles were transected at the inferior border of the thyroid cartilage and resected en bloc with their sternoclavicular attachments. The sternum was transected at the upper border of the third rib, and the clavicles were resected at their midpoints, enabling anterior chest wall removal. The defect was reconstructed with a pectoralis major musculocutaneous flap (PMMC flap). The PMMC flap was harvested from the left chest wall. The flap was elevated along with the pectoralis major muscle, carefully preserving the thoracoacromial artery as its vascular pedicle. It was then rotated superiorly and transposed to the defect to cover the exposed superior mediastinum. The muscle bulk was utilized to obliterate the dead space created by the resection of the sternum and clavicles, while the skin island provided external coverage.

**FIGURE 7:**
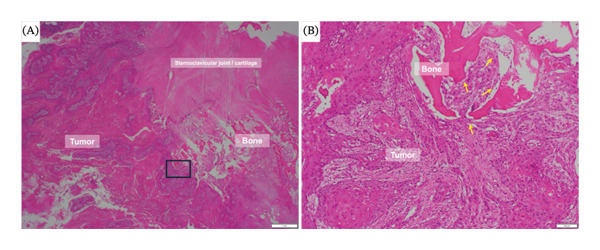
Histopathological findings. Malignant cells with nuclear atypia invading bone, with cortical bone destruction (arrow). The selected area in (A) is shown at higher magnification (× 100) in (B) for clarification.

Histopathological examination revealed disruption of the metastatic lymph node boundary with direct invasion into the adjacent sternal bone and sternoclavicular joint (Figure 7).

**FIGURE 8:**
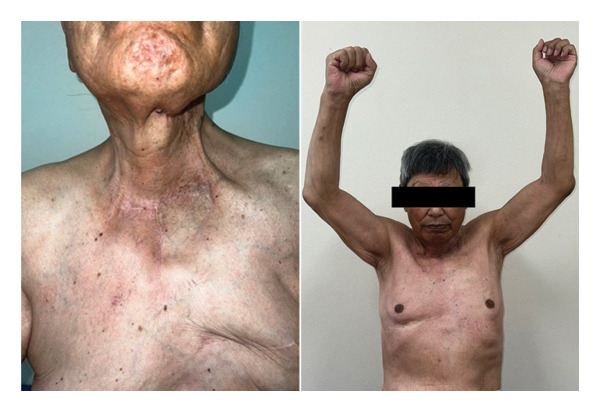
Outpatient follow‐up image. The surgical wound healed well. Although resection of the sternoclavicular joint is generally associated with postoperative shoulder dysfunction, the patient retained full shoulder abduction without functional limitation.

These findings suggested no hematogenous bone metastasis but rather a direct extension of lymph node metastasis into the bone. Surgical margins were histologically negative. The patient had no postoperative complications during hospitalization and remained recurrence‐free for 18 months, with no decline in activities of daily living. In addition, the patient retained full ability to abduct the shoulder (Figure 8).

## 3. Discussion

Several cases have reported suprasternal lymphatic metastasis in PTC, known as “Burns space” metastasis [2–4]. The lymph node in the suprasternal space (LNSS), located between the sternocleidomastoid and sternohyoid muscles, is demarcated superiorly by the intersection of these muscles, inferiorly by the supraclavicular fossa and clavicle, and medially and laterally by the sternohyoid muscle [2]. Anatomically, this region is not adjacent to the aortic arch or brachiocephalic vein. Although the LNSS region is not defined in current head and neck cancer management guidelines, including those of the American Head and Neck Society and the American Academy of Otolaryngology‐Head and Neck Surgery, it may harbor metastasis [5]. While not formally part of Level VI, the LNSS lies in proximity, separated only by the strap muscles, sternothyroid and sternohyoid. Therefore, preoperative assessment is needed. If metastasis is suspected in this region, complete surgical resection should be considered. Sun et al. reported an LNSS metastasis rate of 22.6% in patients with PTC and clinically positive lateral lymph nodes [2]. Additionally, risk factors for LNSS metastasis include primary tumors at the inferior pole and Level IV metastasis [2, 6]. However, the rates of metastasis and risk factors for LNSS involvement in oral SCC have not been established. This report describes a rare case of suprasternal lymphatic metastasis arising from lingual cancer. To our knowledge, this is the first documented case outside of PTC.

Typically, lymphatic drainage from the oral cavity proceeds sequentially through Levels I–III before reaching Level IV [7]. However, after dissection of Levels II–IV, disruption of the normal lymphatic pathways may alter lymphatic drainage patterns, potentially facilitating drainage toward the suprasternal lymph nodes and LNSS.

Moreover, the efferent lymphatic flow from Level VI drains into Levels II–IV [7]. Consequently, neck dissection may cause lymphatic stagnation at Level VI, potentially facilitating metastasis to the adjacent suprasternal lymph nodes and LNSS. However, this mechanism remains speculative.

Given this altered lymphatic drainage pattern, malignancies of the larynx, cervical esophagus, anterior neck, hypopharynx, and thyroid gland similarly metastasize to the suprasternal lymph nodes and LNSS due to postdissection stagnation at Level VI. Another possible cause of metastasis to the suprasternal lymph nodes and LNSS in this case was the low dosage of the radiation therapy delivered to the suprasternal region. Radiation field mapping revealed that the supraclavicular area received only 20–30 Gy (Figure 3), which is lower than the standard prophylactic dose of 50 Gy for subclinical disease, which is required to delay lymph node metastasis [8]. Whether this lower dose contributed to the development of metastasis in the present case remains uncertain.

Furthermore, a substantial secondary finding was the histopathologically confirmed direct tumor extension into the sternum, clavicle, and sternoclavicular joint, an exceedingly rare occurrence in head and neck malignancies. Although beyond the primary scope of this study, these findings underscore the need for aggressive surgical resection when bone invasion is suspected, particularly in the absence of distant metastasis.

Because this is a single case report, the proposed mechanisms involving altered lymphatic drainage after neck dissection and the potential influence of radiation dose should be regarded as hypotheses rather than established explanations. Further studies are needed to clarify their roles in the development of suprasternal lymph node metastasis.

## 4. Conclusion

In addition to PTC, lingual SCC can metastasize to Burns lymph nodes. Careful evaluation of the Burns space is important in patients with a history of neck dissection. Clinicians should highly suspect metastasis in this region when encountering a palpable midline mass or abnormal radiological findings in the Burns space during postoperative surveillance.

## Author Contributions

Writing–original draft preparation: Taisei Yasuda prepared the manuscript. All authors contributed to the acquisition of data for this paper.

## Funding

The authors received no specific funding for this work.

## Disclosure

All authors meet the ICMJE authorship criteria.

## Ethics Statement

The study complied with the Declaration of Helsinki and the current ethical guidelines.

## Consent

The patient reported in this manuscript provided written informed consent for the publication of the case details.

## Conflicts of Interest

The authors declare no conflicts of interest.

## Data Availability

The manuscript has no associated data or the data will not be deposited.
